# Predicting self-rated mental and physical health: the contributions of subjective socioeconomic status and personal relative deprivation

**DOI:** 10.3389/fpsyg.2015.01415

**Published:** 2015-09-22

**Authors:** Mitchell J. Callan, Hyunji Kim, William J. Matthews

**Affiliations:** ^1^Department of Psychology, University of EssexColchester, UK; ^2^Department of Psychology, University of CambridgeCambridge, UK

**Keywords:** personal relative deprivation, subjective socioeconomic status, socioeconomic status, physical health, mental health

## Abstract

Lower subjective socioeconomic status (SSS) and higher personal relative deprivation (PRD) relate to poorer health. Both constructs concern people's perceived relative social position, but they differ in their emphasis on the reference groups people use to determine their comparative disadvantage (national population vs. similar others) and the importance of resentment that may arise from such adverse comparisons. We investigated the relative utility of SSS and PRD as predictors of self-rated physical and mental health (e.g., self-rated health, stress, health complaints). Across six studies, self-rated physical and mental health were on the whole better predicted by measures of PRD than by SSS while controlling for objective socioeconomic status (SES), with SSS rarely contributing unique variance over and above PRD and SES. Studies 4–6 discount the possibility that the superiority of PRD over SSS in predicting health is due to psychometric differences (e.g., reliability) or response biases between the measures.

## Introduction

Extensive epidemiological research has shown that socioeconomic status (SES) is a key determinant of public health. People with higher SES have a better quality of living in terms of life expectancy, subjective wellbeing, and medical history than people lower in SES (Adler and Rehkopf, 2008).

More recently, research has compellingly demonstrated that subjective socioeconomic status (SSS) is also an important predictor of health (e.g., Adler et al., 2000; Operario et al., 2004; Singh-Manoux et al., 2005; for recent reviews, see Euteneuer, 2014; Quon and McGrath, 2014). SSS is defined as a “person's subjective perception of their rank, relative to others, in the socioeconomic hierarchy” (Kraus et al., 2013, p. 138). SSS is most often measured using MacArthur's Scale of Subjective Social Status (Adler et al., 2000), which asks respondents to place themselves on a pictorial “SES ladder” that represents a given society, where those with the highest SES (i.e., the most money, highest education and best jobs) are at the top and those with the lowest SES (i.e., the least money, least education, and worst jobs) are at the bottom.

Over and above indicators of objective SES (e.g., income, education), lower SSS has been shown to predict, for example, poorer self-rated health (Operario et al., 2004), higher risk of strokes (Avendano et al., 2006), and lower quality of sleep (Adler et al., 2000). Lower SSS is also correlated with poorer mental health outcomes, such as higher perceived stress (Senn et al., 2014) and depression (Kraus et al., 2013).

Conceptually related to SSS is personal relative deprivation (PRD), which refers to resentment stemming from the belief that one is deprived of a desired and deserved outcome compared to some referent (for reviews, see Crosby, 1976; Smith et al., 2012). More specifically, in their model of relative deprivation, Smith et al. (2012) defined personal/individual relative deprivation as a process characterized by three steps: (1) an individual makes a social comparison with a given target (e.g., similar others) on a given outcome (e.g., material wealth), (2) a cognitive appraisal leads the individual to believe that she is comparatively disadvantaged, which (3) gives rise to feelings of resentment and dissatisfaction. Like SSS, measures of PRD have been shown to associate with mental and physical health outcomes (see Smith et al., 2012), such as lower self-esteem (Walker, 1999; Callan et al., 2008, 2011), lower subjective well-being (Crosby et al., 1986; Ellaway et al., 2004), poorer self-rated physical health (Osborne et al., 2012), and increased psychological distress (Osborne and Sibley, 2013; Ragnarsdóttir et al., 2013).

SSS and PRD are conceptually related because they both emphasize the importance of an *individual*'s self-perceived *relative* rank within a social hierarchy. They differ, however, in at least two potentially important respects: First, SSS, as measured by the MacArthur visual analog scale, gauges only people's beliefs about their relative social standing. Thus, like Cantril's (1965) self-anchoring scale on which it is based, the MacArthur scale of SSS measures only the cognitive appraisal of one's perceived relative social position and does not directly assess the emotional consequences of believing oneself to be comparatively disadvantaged. This distinction is important because, in their recent meta-analysis of the relative deprivation literature, Smith et al. (2012) found that measures of perceived relative position that included affective judgments (e.g., resentment, dissatisfaction, anger) along with social comparisons more strongly related to internal states and individual behavior across a number of domains than measures that tapped only cognitive appraisals (such as one's relative position on a pictorial “SES ladder”).

SSS and PRD also differ in the underlying comparison processes. The SSS ladder asks people to compare themselves to “all the people in society” in terms of education, money, and jobs, and the position they select is thought to represent a global self-assessment of socio-economic status formed by a “cognitive averaging of standard markers of socioeconomic position [that] is free of psychological biases” (Singh-Manoux et al., 2003, p. 1321; see also Nielsen et al., 2015). Measures of PRD often focus more on specific, local, interpersonal comparisons (e.g., what similar others have) than, for example, one's perceived rank within the national population (Adler et al., 2000) or one's community (Goodman et al., 2001). For instance, Callan et al.'s (2008, 2011) Personal Relative Deprivation Scale (PRDS) was developed to gauge respondents' resentment and dissatisfaction arising from comparing what they have with what similar others have. This focus on social comparisons with similar others was guided by Festinger's (1954) “similarity hypothesis” of social comparison, which suggests that people generally prefer to compare themselves with individuals who are similar to themselves when evaluating their standing on a given outcome, attribute, ability, or opinion (for a more developed analyses of this issue, see Wood, 1989; Suls et al., 2002). In addition, the PRDS does not specify the dimension(s) on which people feel relatively deprived, potentially capturing a broader conception of people's relative social success than one defined in terms of conventional socioeconomic indicators.

One potentially important consequence of the distinction between SSS and PRD is that two people could place themselves on the same ladder rung of the MacArthur SSS scale but have very different experiences of PRD. For example, two professors within the same department having the same salary, years of higher education, years in service, and publication records might report similar SSS (assuming an overall assessment or “cognitive averaging” of these status indicators), but they might not experience the same levels of perceived unfairness and resentment. For example, one may feel resentful on other dimensions (e.g., by having fewer close friends), make material comparisons with a different referent (e.g., a millionaire brother-in-law), or simply have a different affective response to their relative standing in society (e.g., by practicing Buddhism). Such differences in PRD might have consequences for people's physical and mental health over and above where people position themselves on a pictorial ladder representing national SES. Moreover, current explanations for why lower SES affects ill-health emphasize the roles of limited access to resources for maintaining and restoring health and the deleterious effects of physical and social environments associated with low SES (e.g., greater exposure to pathogens and higher levels of crime; see Adler and Snibbe, 2003). The PRD perspective, however, suggests that even those with access to relatively plentiful financial resources (such as our two professors) and who inhabit environments conducive to good health can feel resentful and angry about their lot in life, and those with access to very few resources may not necessarily feel unfairly disadvantaged (see Smith et al., 2012; Smith and Pettigrew, 2014). At the same time, two people might feel equally resentful compared to similar others but put themselves on different ladder rungs of the SSS ladder. This possibility would point to unique effects for SSS in terms of predicting health over and above the potential associations with PRD.

## Overview of current research

Across six studies, we investigated the relative utility of SSS and PRD as correlates of self-reported physical and mental health indicators. Although both constructs have been shown to relate to poorer health outcomes, to our knowledge no research has examined the relative importance of each within the same investigation. Along with measures of objective SES, SSS, and PRD, we measured a broad range of self-reported mental and physical health outcomes, including physical and mental health impairment, depression, negative affect, perceived stress, sleep quality, and physical health complaints. For four of our studies, we included a single item measure of self-rated global health, which is a potent predictor of all-cause mortality across a variety of populations (Idler and Benyamini, 1997; DeSalvo et al., 2006). In Study 6, we compared the test-retest reliability of measures of SSS and PRD, and explored whether changes in SSS and PRD correspond to changes in stress over a 6-week period. In light of the foregoing analysis, these studies ask: is self-rated physical and mental health better predicted by PRD or by SSS?

## Study 1

### Methods

#### Participants

We recruited participants from the USA through Amazon's Mechanical Turk (*N* = 356; see Paolacci et al., 2010; Buhrmester et al., 2011; Shapiro et al., 2013)^1^. Participants were given a nominal payment for completing the online survey. Sample characteristics are shown in Table 1.

**Table 1:** **Sample Characteristics**.

	**Study 1**	**Study 2**	**Study 4**	**Study 5**
*N*	356	397	400	404
*M* age (*SD*)	33.81 (11.74)	32.28 (10.69)	36.12 (11.74)	33.06 (10.53)
**SEX (%)**				
Male	64	67	55	62
Female	36	32	45	38
Unreported	0.3	0.5	0.8	0
**INCOME (%)**				
≤ $15,000	14	9	13	15
$15,001–$25,000	16	15	11	13
$25,001–$35,000	19	15	16	14
$35,001–$50,000	14	20	21	19
$50,001–$75,000	20	23	18	17
$75,001–$100,000	9	9	14	13
$100,001–$150,000	6	7	7	6
>$150,000	2	2	2	4
**EDUCATION (%)**				
Did not finish high school	1	1	1	1
High school graduation	42	42	37	36
College graduation	53	47	47	52
Postgraduate degree	4	10	15	12

#### Procedure and measures

Participants completed the measures outlined below. The order of the first two measures, PRDS and SSS, was counterbalanced between-subjects (with random assignment). The measures employed to assess self-rated mental and physical health followed closely those used by Adler et al. (2000) and Kraus et al. (2013). These measures were presented in a random order after the PRDS and SSS measures. Finally, participants completed measures of objective SES (i.e., income, education) and reported their age and gender.

##### Subjective socioeconomic status

Participants completed MacArthur's Scale of Subjective Social Status (Adler et al., 2000). They were presented with a graphical 10-rung ladder representing “where people stand in the United States,” with the top rung representing the best off, and the bottom rung representing the worst off, in terms of education, money and jobs in the USA. Each participant clicked on the rung to indicate where they thought they stood at that time in their lives, relative to other people in the USA. Higher scores indicate higher SSS.

##### Personal relative deprivation scale

PRD was assessed using Callan et al.'s (2011) five-item PRDS. In the context of research into the link between PRD and gambling (Callan et al., 2008, 2011), the PRDS was developed to gauge people's general perceptions and emotions associated with comparing their outcomes to the outcomes of similar others (“I feel deprived when I think about what I have compared to what other people like me have”; “I feel privileged compared to other people like me”; “I feel resentful when I see how prosperous other people like me seem to be”; “When I compare what I have with what others like me have, I realize that I am quite well off”; “I feel dissatisfied with what I have compared to what other people like me have”). Items were rated using a 6-point scale (1 = *strongly disagree*, 6 = *strongly agree;* items 2 and 4 were reverse scored). Higher scores indicate higher PRD.

##### Self-rated health

Self-rated health was measured using Ware et al. (1996) Short Form-12 (SF-12) questionnaire, a widely used questionnaire that produces separate component summary scores for mental and physical health impairments. The items for the physical health component relate to how one's health affects everyday functioning (e.g., “climbing several flights of stairs”), whereas the items for the mental health component relate to one's mood and emotional problems over the previous 4 weeks (e.g., “have you felt downhearted and blue”). The 12 items were transformed and scored according to the standard procedure detailed by Ware et al.'s (1995) manual. This scoring procedure resulted in two uncorrelated mental and physical health components, each with scores ranging from 0 to 100 (higher values indicate greater mental and physical health *impairments*). Following Kraus et al.'s (2013) analysis strategy, we also examined the first item from the SF-12 as a separate measure of global health (“In general, my health is,” which was rated from 1 = *excellent* to 5 = *poor*). This item was rescaled so that higher values indicate better overall global health.

##### Depression

Depression was measured using the 20-item Center for Epidemiological Studies Depression Scale (CES-D; Radloff, 1977). Participants rated how often they experienced a number of difficulties over the previous week (e.g., “I was bothered by things that usually don't bother me”; “I felt depressed”). The items were rated using a 4-point scale ranging from 1 = *rarely or none of the time* (*less than 1 day*) to 4 = *most or all of the time* (*5–7 days*). Higher scores on the CES-D indicate greater depression.

##### General negative affect

General negative affect was measured using the 10-item Negative Affect subscale of the Positive and Negative Affect Scale (PANAS; Watson et al., 1988). Participants rated the extent to which they felt the given emotions (i.e., irritable, distressed, ashamed, upset, nervous, guilty, scared, hostile, jittery, afraid) in general on a 5-point scale (1 = *very slightly or not at all* to 5 = *extremely*).

##### Objective socioeconomic status

Following Kraus et al. (2013), to assess objective SES, participants reported their annual household income before taxes by selecting from eight ranges of incomes (1 ≤ $15,000, 2 = $15,001–$25,000, 3 = $25,001–$35,000, 4 = $35,001–$50,000, 5 = $50,001–$75,000, 6 = $75,001–$100,000, 7 = $100,001–$150,000, 8 ≤ $150,000). Participants also indicated the highest level of their educational attainment among four choices (1 = *did not finish high school*, 2 = *high school graduation*, 3 = *college graduation*, 4 = *postgraduate degree*).

### Results

#### Correlation and multiple regression analyses

Shown in Table 2, PRDS and SSS were moderately and significantly negatively correlated and, replicating previous research, SSS correlated significantly with all of the physical and mental health measures, such that lower SSS was generally related to worse health outcomes. A similar pattern of correlations emerged for PRDS, such that, with the exception of physical health impairment, higher PRD significantly related to worse health outcomes.

**Table 2:** **Descriptive statistics and intercorrelations for measures in Study 1**.

**Measures**	***M* (*SD*)**	**1**	**2**	**3**	**4**	**5**	**6**	**7**	**8**	**9**
1. SSS	4.67 (1.76)	–								
2. PRDS	3.21 (0.99)	−0.52^**^	(0.83)							
3. Income	3.72 (1.87)	0.58^**^	−0.32^**^	–						
4. Education	2.60 (0.60)	0.32^**^	−0.15^**^	0.26^**^	–					
5. Global health	3.35 (0.97)	0.28^**^	−0.28^**^	0.20^**^	0.11^*^	–				
6. Physical health impairment	48.81 (7.83)	−0.12^*^	0.10	−0.13^*^	−0.05	−0.56^**^	(0.59)			
7. Mental health impairment	53.09 (11.56)	−0.38^**^	0.48^**^	−0.26^**^	−0.09	−0.40^**^	0.00	(0.71)		
8. Depression	1.89 (0.39)	−0.18^**^	0.38^**^	−0.16^**^	−0.11^*^	−0.29^**^	0.19^**^	0.68^**^	(0.79)	
9. Negative Affect	1.57 (0.72)	−0.23^**^	0.38^**^	−0.22^**^	−0.07	−0.32^**^	0.17^**^	0.70^**^	0.77^**^	(0.94)

*SSS, Subjective Socioeconomic Status; PRDS, Personal Relative Deprivation Scale. When applicable, alpha reliabilities are presented in parentheses along the diagonal. Correlations with mental and physical health impairment are N = 348 due to missing values*.

* *p < 0.05*.

** *p < 0.01*.

We performed a series of multiple regression analyses to test the unique contributions of PRD and SSS to the prediction of the physical and mental health indicators while also controlling for income and education^2^. Shown in Table 3, PRD accounted for significant incremental variance in global health and all the mental health variables, whereas SSS was only a unique significant predictor of global health and mental health impairment^3^. Neither variable accounted for significant unique variance in physical health impairment.

**Table 3:** **Simultaneous multiple regression analyses predicting heath indicators from SSS, PRD, income, and education across studies**.

	**SSS**				**PRD**				**Income**				**Education**			
	***b***	**β**	***sr^2^***	***GDW***	***b***	**β**	***sr^2^***	***GDW***	***b***	**β**	***sr^2^***	***GDW***	***b***	**β**	***sr^2^***	***GDW***
**STUDY 1 (***N* = 356**)**																
Global Health	0.09	0.15^*^	0.012	0.04	−0.18	−0.18^**^	0.024	0.047	0.02	0.04	0.001	0.015	0.04	0.03	0.001	0.005
Phys. Health Impair	−0.14	−0.03	0.001	0.006	0.39	0.05	0.002	0.004	−0.41	−0.10	0.006	0.011	−0.12	−0.01	0.000	0.001
Ment. Health Impair	−0.98	−0.15^*^	0.012	0.067	4.58	0.39^**^	0.111	0.162	−0.36	−0.06	0.002	0.024	0.68	0.04	0.001	0.003
Depression	0.02	0.08	0.003	0.013	0.16	0.39^**^	0.112	0.124	−0.01	−0.06	0.002	0.009	−0.04	−0.06	0.003	0.006
Negative Affect	0.01	0.03	0.001	0.019	0.26	0.36^**^	0.095	0.115	−0.13	−0.13^*^	0.011	0.023	0.01	0.01	0.000	0.001
**STUDY 2 (***N* = 397**)**																
Global Phys. Health	0.02	0.04	0.001	0.022	−0.21	−0.22^**^	0.039	0.059	0.07	0.13^*^	0.013	0.031	0.15	0.11^*^	0.011	0.018
Perceived Stress	−0.02	−0.05	0.002	0.032	0.40	0.52^**^	0.213	0.244	0.01	0.03	0.001	0.009	−0.001	−0.00	0.000	0.003
Sleep Quality	−0.01	−0.01	0.000	0.011	0.23	0.32^**^	0.08	0.092	−0.001	−0.01	0.000	0.005	−0.03	−0.03	0.001	0.003
Sleep-Onset Latency	0.01	0.01	0.000	0.008	0.45	0.25^**^	0.05	0.06	−0.08	−0.07	0.004	0.012	−0.10	−0.04	0.001	0.003
**STUDY 3 (***N* = 366**)**																
Global Phys. Health	0.16	0.20^**^	0.028	0.052	−0.26	−0.19^**^	0.029	0.05	0.003	0.01	0.000	0.006	0.01	0.03	0.001	0.003
Perceived Stress	−0.01	−0.03	0.001	0.026	0.37	0.48^**^	0.183	0.21	−0.01	−0.03	0.001	0.01	0.004	0.01	0.000	0.000
Physical Complaints	0.03	0.08	0.004	0.004	0.14	0.22^**^	0.039	0.043	−0.02	−0.15^*^	0.019	0.022	−0.02	−0.07	0.005	0.004
**STUDY 4 (***N* = 400**)**																
Perceived Stress	−0.07	−0.15^*^	0.01	0.067	0.11	0.30^**^	0.059	0.105	−0.06	−0.12^*^	0.009	0.039	−0.002	−0.00	0.000	0.01
**STUDY 5 (***N* = 404**)**																
Resentment	−0.09	−0.18^**^	0.015	0.092	0.17	0.32^**^	0.056	0.126	−0.03	−0.08	0.004	0.037	−0.07	−0.06	0.003	0.009
Global Phys. Health	0.05	0.06	0.001	0.033	−0.24	−0.27^**^	0.039	0.067	0.03	0.04	0.001	0.015	0.22	0.11^*^	0.01	0.014
	Δ SSS				Δ PRD				Δ Income				Δ Education			
**STUDY 6 (***N* = 118**)**																
Change in Stress	−0.01	−0.02	0.001	0.001	0.27	0.38^**^	0.141	0.140	−0.02	−0.09	0.008	0.006	−0.02	−0.04	0.002	0.001

*SSS, Subjective Socioeconomic Status; PRD, Personal Relative Deprivation; Δ, change; b, unstandardized regression coefficient. β, standardized regression coefficient; sr^2^, semi-partial correlation squared; GDW, general dominance weight. GDWs for each criterion sum to the total model R^2^. Higher values for sleep measures indicate more disturbed sleep. Otherwise, higher values indicate more of each construct*.

* *p < 0.05*.

** *p < 0.01*.

Across our studies, we supplemented our multiple regression analyses with dominance analysis (Azen and Budescu, 2003; Azen, 2013), which is a method of variance partitioning that establishes the relative contribution a predictor makes to a criterion by itself and in combination with other predictors by comparing its incremental validity (semi-partial correlation squared, *sr*^2^) across all possible regression submodels that involve that predictor. Dominance analysis helps to overcome the problems associated with establishing relative importance with correlated predictors (Azen, 2013). General dominance weights (GDW; see Table 3) represent the average incremental contribution each predictor makes across all possible submodels; they always sum to the overall model *R*^2^ for a given criterion, which allows for a rank-ordering of the average contribution of each predictor to a criterion by itself and when taking all other predictors into account. Dominance analyses were performed using the *yhat* package for R (Nimon et al., 2013; see also Nimon and Oswald, 2013). As with our multiple regression analyses, these analyses included PRD, SSS, income, and education as predictors of the health measures we employed.

Generalization of the observed rank-ordering of GDWs between PRD and SSS were determined across our studies using bootstrapped resampling analyses (1000 resamples) suggested by Azen (2013). These analyses yield a measure called “reproducibility” (expressed as a proportion), which represents “how often one can expect each dominance relationship observed in the (original or parent) sample to hold in the population” (Azen, 2013, p. 51). For example, a reproducibility rate of 90% for PRD generally dominating SSS in the prediction of, say, negative affect indicates that this dominance relationship is reproduced in 90% of the bootstrap samples. Based on simulation studies, Azen (2013) suggested that reproducibility rates greater than 70% indicate that one can have high confidence that the dominance relationship observed in the sample holds in the population. Shown in Table 3, PRD generally dominated SSS in predicting mental health, depression, and negative affect (all reproducibility values >99.3%). Neither PRD nor SSS dominated each other in terms of predicting self-rated health and physical health impairment (reproducibility values for PRD dominating SSS of 61.80 and 57.70%, respectively).

#### Mediation analyses

Researchers have found that psychosocial risk factors (e.g., negative affect, perceived stress) mediate the relations between SSS and physical and mental health (e.g., Singh-Manoux et al., 2003; Operario et al., 2004; Cundiff et al., 2013; Senn et al., 2014). According to this hypothesis, the link between lower SSS and poorer health reflects the negative physiological consequences (e.g., stress, negative affect) of perceiving oneself as relatively low in status. Consistent with this view, Kraus et al. (2013) found that, controlling for objective SES, “chronic negative affect” (which they operationally defined as scores on the CES-D scale) mediated the relations between SSS and self-rated global health and SSS and mental health impairment. Using Preacher and Hayes's (2008) bootstrapping procedure for testing indirect effects, we performed Kraus et al.'s analyses with SSS but included PRD as a covariate (along with income and education). These analyses revealed bias-corrected and accelerated 95% confidence intervals (95% BCa CI) of −0.03 and 0.01 (total effect = 0.08; indirect effect = −0.01, *SE* = 0.01) and −0.28 and 0.81 (total effect = −0.98; indirect effect = 0.27, *SE* = 0.27) for tests of the indirect effects of SSS on global health and SSS on mental health impairments through chronic negative affect, respectively. These results show that, controlling for PRD, income, and education, chronic negative affect is not a significant mediator of the links between SSS and global health and SSS and mental health impairment. Analyses testing the indirect effects of PRD on global health (95% BCa CI of −0.15 and −0.04; total effect = −0.18; indirect effect = −0.09, *SE* = 0.03) and PRD on mental health impairment (95% BCa CI of 1.84 and 3.68; total effect = 4.58; indirect effect = 2.70, *SE* = 0.46) through chronic negative affect while controlling for SSS, income, and education revealed chronic negative affect as a significant mediator of these relations. It is important to note, however, that directionality cannot be determined from these analyses given the cross-sectionality of the data (e.g., negative affect could cause both PRD and global health).

### Discussion

One potential limitation of Study 1 is that PRD might have more strongly correlated with the outcome variables we employed than SSS because the PRDS directly gauges negative feeling states more than the “SES ladder” measure does. Put differently, the affectively-laden items of the PRDS (e.g., “I feel resentful…”) might have led to stronger correlations with the mental health measures than SSS because these measures also include items that are similarly affectively toned. Our aim in Studies 2 and 3, then, was to extend our Study 1 findings to different health-related outcomes that do not all explicitly ask participants to report their negative affect. Along with PRD, SSS, income, and education, we measured global physical health, perceived stress, sleep quality, and sleep-onset latency in Study 2, and global physical health, perceived stress, and physical health complaints (e.g., headaches, sore throat) in Study 3. The global health measure and the sleep measures do not ask participants to self-report their negative affect (e.g., “During the past month, how long (in minutes) has it usually taken you to fall asleep each night?” for sleep onset latency) and therefore should not overlap with the PRDS any more than with the SES ladder in terms of common self-descriptors or related response biases. Importantly, measures of sleep quality and physical health complaints have been employed in previous research on the association between SSS and health (e.g., Adler et al., 2000; Hamad et al., 2008; Cundiff et al., 2013; Jarrin et al., 2013; Quon and McGrath, 2014; Thompson et al., 2014). In addition, in Study 3 we employed a sample of participants living in the United Kingdom to test the generalizability of our findings outside of the American context.

## Study 2

### Methods

#### Participants

Participants from the USA were recruited as in Study 1 (*N* = 397). Sample characteristics are shown in Table 1.

#### Procedure and measures

Participants first completed the SSS measure and PRDS in a random order. Next, the following measures were presented in a random order.

##### Perceived stress

Participants completed the 10-item Perceived Stress Scale (Cohen and Williamson, 1988). They indicated how often they experienced various thoughts over the last month (e.g., “In the last month, how often have you felt nervous and ‘stressed’?” (1 = *never* to 5 = *very often*). Higher scores indicate greater perceived stress.

##### Sleep quality and sleep-onset latency

To assess quality of sleep, participants completed the sleep quality and sleep-onset latency subscales of the Pittsburgh Sleep Quality Index (Buysse et al., 1989). The subscales involved three questions assessing the subjective quality of sleep, average time to fall asleep, and frequency of trouble falling asleep during the past month. Higher scores on the subscales indicate *worse* quality of sleep and longer sleep-onset latency.

##### Self-rated global physical health

Participants reported their general physical health status using a single-item (“In general, would you say your physical health is:”) with a 5-point scale (1 = *excellent* to 5 = *poor*). This item was rescaled so higher values indicate better global physical health.

##### Objective socioeconomic status

We measured annual household income and educational attainment as in Study 1 (see Table 1).

### Results

#### Correlation and multiple regression analyses

Shown in Table 4, PRDS and SSS were again moderately and significantly negatively correlated. Both SSS and PRD correlated significantly with global physical health, perceived stress, sleep quality, and sleep-onset latency in the expected directions.

**Table 4:** **Descriptive statistics and intercorrelations for measures in study 2**.

**Measures**	***M* (*SD*)**	**1**	**2**	**3**	**4**	**5**	**6**	**7**	**8**
1. SSS	4.88 (1.63)	–							
2. PRDS	3.12 (1.00)	−0.45^**^	(0.84)						
3. Income	3.94 (1.74)	0.48^**^	−0.32^**^	–					
4. Education	2.65 (0.66)	0.29^**^	−0.16^**^	0.19^**^	–				
5. Global physical health	3.22 (0.94)	0.24^**^	−0.30^**^	0.25^**^	0.18^**^	–			
6. Stress	1.73 (0.76)	−0.27^**^	0.54^**^	−0.16^**^	−0.10	−0.38^**^	(0.91)		
7. Sleep quality	1.22 (0.74)	−0.17^**^	0.33^**^	−0.12^*^	−0.08	−0.43^**^	0.49^**^	–	
8. Sleep latency	2.19 (1.77)	−0.15^**^	0.28^**^	−0.16^**^	−0.09	−0.23^**^	0.45^**^	0.59^**^	(0.82)

*SSS, Subjective Socioeconomic Status; PRDS, Personal Relative Deprivation Scale. When applicable, alpha reliabilities are presented along the diagonal. Higher values for the sleep measures represent more disturbed sleep*.

* *p < 0.05*.

** *p < 0.01*.

Separately for each health outcome measure, we regressed global physical health, perceived stress, sleep quality, and sleep-onset latency onto SSS, PRD, income, and education. Shown in Table 3, PRD uniquely predicted each of the health variables, whereas SSS was not a significant predictor in any of the analyses. Dominance analyses showed that PRD was generally dominant over SSS in the prediction of each of the criterion variables we examined (all reproducibility values >96%).

#### Mediation analyses

In Study 1, following previous research using the social ladder measure of SSS (e.g., Cundiff et al., 2013; Kraus et al., 2013), we examined chronic negative affect as one psychosocial mediator of the relation between perceived social position (SSS and PRD) and adverse health outcomes. Given the well-established links between stress and disturbed sleep (e.g., Kashani et al., 2012) and poorer physical health (e.g., Cohen et al., 2007), we explored whether PRD and/or SSS relate to poorer sleep quality and general physical health through perceived stress. Bootstrapped mediation analyses revealed that, while controlling for SSS, income, and education, perceived stress mediated the relations between PRD and self-rated physical health (95% BCa CI of −0.21 and −0.09; total effect = −0.21; indirect effect = −0.15, *SE* = 0.03), sleep quality (95% BCa CI of 0.11 and 0.21; total effect = 0.23; indirect effect = 0.16, *SE* = 0.02), and sleep-onset latency (95% BCa CI of 0.28 and 0.53; total effect = 0.45; indirect effect = 0.40, *SE* = 0.06). Similar analyses but with SSS as the exogenous variable and PRD, income, and education as covariates revealed no significant indirect effects through perceived stress (indirect effects = 0.01, −0.01, and −0.02, respectively; all 95% BCa CIs contained zero).

## Study 3

### Methods

#### Participants

Participants from the United Kingdom (*N* = 366; *M*_age_ = 33.55, *SD*_age_ = 11.76; % women = 49%) were recruited through either Prolific Academic (prolificacademic.co.uk; *N* = 191) or CrowdFlower (crowdflower.com; *N* = 175), which are online participant recruitment platforms similar to Amazon's Mechanical Turk. Data was collected simultaneously across the two platforms.

#### Procedure and measures

Participants first completed the SSS measure for a UK context (“Please click on the rung where you think you stand at this time in your life, relative to other people in the United Kingdom”) and PRDS in a random order. Next, participant completed the following measures:

##### Perceived stress

Participants completed the 10-item Perceived Stress Scale (Cohen and Williamson, 1988) used in Study 2.

##### Self-rated global physical health

Participants reported their general physical health status using a single-item (“In general, would you say your physical health is:”) with a 7-point scale (1 = *excellent* to 7 = *very poor*). This item was rescaled so higher values indicate better global physical health.

##### Physical health complaints

Participants reported the extent to which they experienced 20 physical problems (*running nose, congested nose, coughing, out of breath, chest pains, racing heart, insomnia or difficulty sleep, upset stomach, indigestion, abdominal pain, diarrhea, tightness in chest, back pains, headaches, feeling pressure in head, dizziness, feel faint, sore throat, nausea, sweat even in cold weather*) using a scale ranging from 1 (*have never or almost never experienced the symptom*) to 5 (*more than once a week*). These 20 physical health complaints were taken from the Pennebaker Inventory of Limbic Languidness (PILL; Pennebaker, 1982). Responses were averaged across items and higher scores indicate more frequent physical health complaints.

##### Objective socioeconomic status

We measured annual household income using an 18-point ordinal scale with values ranging from 1 (less than £5000) to 18 (£85,001 and above), with each option spanning £4999 (*M* = 6.87, *SD* = 4.17). Because the measure of educational attainment we used in our previous studies does not map on well to the UK educational context, we asked participants to report “the number of years of formal education you have achieved since the age of 16 (full-time equivalent)” (*M* = 4.82, *SD* = 2.92)^4^.

### Results

#### Correlation and multiple regression analyses

Shown in Table 5, PRDS and SSS were again moderately and significantly negatively correlated. PRD correlated significantly with self-rated global physical health, perceived stress, and physical health complaints in the expected directions. SSS correlated significantly with perceived stress and global physical health, which confirms Singh-Manoux et al.'s (2003) findings from a sample of British participants.

**Table 5:** **Descriptive statistics and intercorrelations for measures in Study 3**.

**Measures**	***M* (*SD*)**	**1**	**2**	**3**	**4**	**5**	**6**	**7**
1. SSS	5.00 (1.67)	–						
2. PRDS	3.04 (1.00)	−0.43^**^	(0.82)					
3. Income	6.87 (4.17)	0.38^**^	−0.26^**^	–				
4. Education	4.82 (2.91)	0.17^**^	−0.04	−0.05	–			
5. Perceived stress	2.85 (0.77)	−0.24^**^	0.49^**^	−0.16^**^	−0.01	(0.91)		
6. Physical complaints	2.15 (0.62)	−0.08	0.23^**^	−0.17^**^	−0.06	0.46^**^	(0.91)	
7. Global physical health	4.64 (1.35)	0.29^**^	−0.28^**^	0.13^*^	0.07	−0.37^**^	−0.43^**^	–

*SSS, Subjective Socioeconomic Status; PRDS, Personal Relative Deprivation Scale. When applicable, alpha reliabilities are presented along the diagonal. Higher values indicate more of each construct*.

* *p < 0.05*.

** *p < 0.01*.

Shown in Table 3, multiple regression analyses showed that PRD uniquely predicted global physical health, perceived stress, and physical health complaints while controlling for SSS, income, and education, whereas SSS was a significant predictor of only global physical health. Dominance analyses showed that PRD was generally dominant over SSS in the prediction of perceived stress and physical health complaints (both reproducibility values were >99.8%), whereas neither SSS nor PRD were generally dominant in terms of predicting global physical health (reproducibility value for PRD dominating SSS of 52.8%).

#### Mediation analyses

Following our approach in Study 2, we employed bootstrapped mediation analyses (10,000 resamples) to test the mediating role that perceived stress plays in the relations between PRD and physical health and SSS and physical health while controlling for each other and income and education. These analyses revealed that, while controlling for SSS, income, and education, perceived stress mediated the relations between PRD and global physical health (95% BCa CI of −0.27 and −0.12; total effect = −0.24; indirect effect = −0.19, *SE* = 0.04), and physical health complaints (95% BCa CI of 0.10 and 0.18; total effect = 0.13; indirect effect = 0.14, *SE* = 0.02). Similar analyses with SSS controlling for PRD, income, and education revealed no significant indirect effects through perceived stress for either global physical health (95% BCa CI of −0.02 and 0.04; total effect = 0.18; indirect effect = 0.01, *SE* = 0.01) or physical health complaints (95% BCa CI of −0.02 and 0.02; total effect = 0.02; indirect effect = −0.01, *SE* = 0.01).

## Study 4

Despite the apparent superiority of PRD over SSS in predicting self-rated mental and physical health across Studies 1–3, one issue is that our findings might simply reflect differences in the psychometric properties of the measures we used. All else being equal, the internal reliability of a measure increases as the number of items increase (Carmines and Zeller, 1979). Thus, the five-item PRDS might be more reliable—or at least internally consistent—than the single-item SES ladder simply because it has more items. Further, the PRDS and SES ladder measures differ in the total number of scale points used within the response scales (6 vs. 10, respectively), which can also affect the accuracy of measures (Krosnick and Presser, 2010).

We addressed this issue across Studies 4–6. In Study 4, along with the SES ladder measure of SSS we used in Studies 1–3, we used a single-item from the PRDS and asked participants to rate their agreement on a 10-point scale (which matches the 10-point scale of the SES ladder measure). Given its theoretical importance in the links between PRD and health and SSS and health, we zeroed in on perceived stress as our single criterion variable in Study 4.

### Methods

#### Participants

Participants from the USA were recruited as in Studies 1 and 2 (*N* = 400). Sample characteristics are shown in Table 1.

#### Procedure and measures

Participants first completed the visual analog SSS measure and a single-item PRDS in a random order. They then completed a perceived stress scale and provided their education and annual household income.

##### SSS and PRDS

Participants completed the SES ladder measure as in Studies 1 and 2. For the measure of PRD, participants completed a single-item from Callan et al.'s (2011) PRDS: “When I compare what I have with what others like me have, I realize that I am quite well off,” which was rated on a scale from 1 (*very strongly disagree*) to 10 (*very strongly agree*). We selected this item because, unlike other items from the larger PRDS (but like the SSS measure), it does not ask participants to rate how they feel about their relative standing. Crucially, however, the comparative target is “others like me” vs. the national population of the USA as for the SSS measure. To be consistent with the interpretation of the PRDS in Studies 1–3, we reverse scored this item so that higher values indicate *more* PRD. As before, higher values for the SSS measure indicate a higher subjective relative standing.

##### Perceived stress

Participants completed the four-item Perceived Stress Scale (Cohen and Williamson, 1988): “In the last month, how often have you felt that you were unable to control the important things in your life?”; “In the last month, how often have you felt confident about your ability to handle your personal problems?”; “In the last month, how often have you felt that things were going your way?”; and “In the last month, how often have you felt difficulties were piling up so high that you could not overcome them?.” Items were rated on a 5-point scale (1 = *never*, 5 = *very often*), and higher scores indicate greater perceived stress.

##### Objective socioeconomic status

We measured annual household income and educational attainment as in Studies 1 and 2 (see Table 1).

### Results and discussion

Shown in Table 6, both the single-item PRDS and SSS correlated significantly with perceived stress. Shown in Table 3, multiple regression analyses regressing perceived stress onto PRDS, SSS, income, and education showed that although both PRDS and SSS accounted for significant incremental variance in stress, PRDS accounted for more unique variance in, and was the generally dominant predictor of, perceived stress (reproducibility value of 90%).

**Table 6:** **Descriptive statistics and intercorrelations for measures in Study 4**.

**Measures**	***M* (*SD*)**	**1**	**2**	**3**	**4**	**5**
1. SSS	5.01 (1.81)	–				
2. Single-item PRDS	5.09 (2.16)	−0.57^**^	–			
3. Stress	2.55 (0.83)	−0.39^**^	0.42^**^	–		
4. Income	4.01 (1.85)	0.60^**^	−0.34^**^	−0.31^**^	–	
5. Education	2.76 (0.71)	0.40^**^	−0.28^**^	−0.19^**^	0.35^**^	–

*SSS, Subjective Socioeconomic Status; PRDS, Personal Relative Deprivation Scale. Higher values indicate more of each construct*.

** *p < 0.01*.

## Study 5

In Study 4, perceived stress was better predicted by a single-item from the PRDS than by the single-item SES ladder measure. Nonetheless, the content of the questions was not the same across the two measures (i.e., perceived privilege vs. position on an SES ladder) and response scales were different (i.e., visual vs. verbal). In Study 5 we addressed the issue of reliability across SSS and PRD measures in another way: participants rated their SSS and PRD using the *same* items and response scales that differed only in the targets for comparison (i.e., compared to people in the USA vs. similar others). Assessing SSS and PRD using essentially the same items also allowed us to address an additional potential limitation of our first three studies: Given that people higher in general negative affectivity tend to report their health status as being worse than it actually is (Watson and Pennebaker, 1991), the PRDS (vs. SES ladder) might better predict health outcomes because it asks people to rate their negative affect along with their perceived relative disadvantage (i.e., affect and social comparisons are, by design, conflated within the PRDS measure). Along with self-rated global physical health, In Study 5 we measured people's resentment about their social standing *separately* from their beliefs about their comparative disadvantage. This approach allowed us to examine resentment as a mediator of the relations between SSS and health and PRD and health.

Do measures of SSS and PRD gauge different but related constructs or essentially the same underlying construct? In Study 5 we began to answer this question by asking participants to report the social comparison targets that came to mind for them when they rated their relative standing compared to people in the USA and similar others. We have argued that one key difference between SSS and PRD is the specificity of the social comparisons people use to derive their perceived relative disadvantage. Thus, if our measure of PRD gauges more local, specific social comparisons than the measure of SSS, then people should report making more specific social comparisons when they rated their relative disadvantage compared to “similar others” than “people in the USA.”

### Methods

#### Participants

Participants from the USA were recruited as in Study 1 (*N* = 404). Sample characteristics are shown in Table 1.

#### Procedure and measures

Participants completed the following measures in order:

##### Comparisons with people in the USA and similar others

Using a single-item measure of SSS from previous research (which has been shown to correlate with self-rated health; Wolff et al., 2010), we asked participants to rate their relative standing compared with people in the USA:

Please think about where you stand at this time in your life compared with people in the United States. Some people in the United States are better off—they have more money, more education, and better jobs. Other people in the United States are worse off—they have less money, less education, and worse jobs.

How do you think your current standing in life compares with that of people in the United States?

Participants provided their response using a scale ranging from 1 (*I'm very much worse off*) to 9 (*I'm very much better off*). Each scale point included a verbal description (*very much, much, somewhat, slightly*, and *about the same* across “worse off” to “better off”). The item assessing perceived relative standing compared to *similar others* was exactly the same except for the comparison target. Here, participants rated their current standing compared with “people who are like you.” These two items were counterbalanced in a random order. To provide a consistent interpretation of PRD across our studies, the item for “compared to people who are like you” (hereafter referred to as PRD) was reverse scored so that higher values indicate greater perceived relative disadvantage compare with similar others.

##### Resentment

Participants rated the extent to which they felt *dissatisfied, resentful, satisfied*, and *angry* when they thought about where they stood at this time in their lives (cf. Callan et al., 2008; Osborne et al., 2012). These items were rated on a scale ranging from 1 (*very slightly or not at all*) to 5 (*extremely*). The *satisfied* item was reversed score and the items were averaged to form one measure of resentment; higher values indicate more resentment.

##### Self-rated global physical health

Participants reported their general physical health status using a single-item (“In general, would you say your physical health is:”) with a 7-point scale (1 = *excellent* to 7 = *very poor*). This item was rescaled so higher values indicate better global physical health.

##### Open-ended responses.

For both the SSS and PRD measures, we asked participants to report, in an open-ended comment box, who came to mind for them when they were rating their relative standing on the previous pages (“We'd like to know who came to mind when you were answering this question. With whom did you compare yourself?”). They were given separate comment boxes for the general, American comparisons and specific, “like you” comparisons, and these were presented in a random order across participants.

##### Objective socioeconomic status

We measured annual household income and educational attainment as in Study 1 (see Table 1).

### Results

#### Correlation and multiple regression analyses

Shown in Table 7, both the SSS and PRD items correlated significantly with resentment and self-rated physical health. Shown in Table 3, multiple regression analyses showed that both measures were significant predictors of resentment over and above each other and income and education, but only PRD uniquely predicted self-rated physical health. Dominance analyses showed that PRD was generally dominant over SSS for the prediction of both resentment and self-rated physical health (both reproducibility values > 86%).

**Table 7:** **Descriptive statistics and intercorrelations for measures in Study 5**.

**Measures**	***M* (*SD*)**	**1**	**2**	**3**	**4**	**5**	**6**
1. SSS (USA)	5.00 (1.66)	–					
2. PRD (similar others)	5.19 (1.54)	−0.66^**^	–				
3. Income	3.90 (1.95)	0.53^**^	−0.39^**^	–			
4. Education	2.75 (0.67)	0.24^**^	−0.09	0.29^**^	–		
5. Resentment	2.23 (0.77)	−0.45^**^	0.47^**^	−0.31^**^	−0.15^**^	(0.80)	
6. Global Physical Health	4.75 (1.37)	0.28^**^	−0.33^**^	0.21^**^	0.16^**^	−0.45^**^	–

*When applicable, alpha reliabilities are presented along the diagonal. Higher values for “compared to similar others” indicate greater perceived relative disadvantage. Otherwise, higher values indicate more of each construct*.

** *p < 0.01*.

#### Mediation analyses

Following our approach in our previous studies, we employed bootstrapped mediation analyses (10,000 resamples) to test the mediating role that resentment plays in the relations between perceived comparative (dis)advantage for SSS and self-rated health and PRD and self-rated health while controlling for each other and income and education. These analyses revealed that, while controlling for SSS, income, and education, resentment mediated the relation between PRD and global physical health (95% BCa CI of −0.16 and −0.06; total effect = −0.24; indirect effect = −0.11, *SE* = 0.03). Similar analyses with SSS controlling for PRD, income, and education revealed that the small and non-significant total effect SSS had on self-rated health was also mediated by resentment (95% BCa CI of 0.02 and 0.11; total effect = 0.05; indirect effect = 0.06, *SE* = 0.02).

#### Content analysis of social comparison targets

Two raters coded participants' open-ended responses to the questions of who came to mind when they were answering the SSS and PRD items. The responses were coded into six categories: *past and/or present friends* (“I thought about my best friend”), *family* (“I was thinking about my siblings”), *co-workers/colleagues* (“I thought about my peers at work—the other middle managers on our team”), *neighbors and members of local community* (“my neighbors”), *classmates from college and/or school* (“I thought of my classmates with whom I graduated college”), *peers and/or acquaintances* (“I thought about the people who go to the same church that I do”), *general social comparisons* (“I compared myself to what I read about as the median earner in this country”; “An average American making an average income”), and *no social comparisons* (“no one came to mind”). For comparison, a separate sample of 95 participants (*M*_age_ = 32.22, *SD*_age_ = 8.55; 55% male) recruited through MTurk answered the same open-ended question after completing Callan et al.'s five-item PRDS in isolation; these responses were coded in the same way as the responses to the SSS and PRD items in the current study. The mean inter-rater agreement (Cohen's kappa) across categories was 0.83, and differences were resolved through discussion.

Shown in Table 8, the overall pattern was that participants typically mentioned significantly more specific social comparison targets for the PRD measures (e.g., friends, co-workers) than the SSS measure, whereas they mentioned more general comparison targets (e.g., median income in America) for the SSS measure than the PRD measures (participants often provided more than one comparative referent, hence why these percentages sum to over 100% across rows).

**Table 8:** **Percentages of responses to the open-ended questions of who the participants compared themselves with when rating their standing compared to others in the USA and people like them (Study 5)**.

	**Friends Friends**	**Family members**	**Co-Workers**	**Neighbors/Community**	**Classmates**	**Peers and acquaint**	**Others in general**	**No comparison**
**MEASURE**								
Compared to American society (SSS)	5_a_	5_a_	4_a_	9_a_	2_a_	9_a_	73_a_	7_a_
Compared to similar others (PRD)	37_b_	9_b_	13_b_	7_a_	10_b_	12_a_	35_b_	7_a_
Personal relative deprivation scale	46_b_	20_c_	18_b_	13_a_	10_b_	10_a_	17_c_	4_a_

*SSS, Subjective Socioeconomic Status; PRD, Personal Relative Deprivation. Values shown are percentages of responses within samples. Values that do not share subscripts within columns are significantly different at p < 0.05. Responses to the open-ended questions following the Personal Relative Deprivation Scale were obtained from a separate sample of participants (N = 95). Significance tests for the differences of proportions of responses between SSS and PRD were conducted using the McNemar's Test; comparisons of responses to the Personal Relative Deprivation Scale with SSS and PRD were conducted using the z-ratio for significant differences between independent proportions*.

## Study 6

In Study 5, we showed that using the same items and response scale, resentment and global physical health were better predicted by participants' perceived relative position compared to similar others than compared to people in the USA. Moreover, participants thought of different social comparison targets while completing the measures, which reflected the relative specificity of the targets given in the measures (general vs. similar others). The consistently superior predictive validity of PRD over SSS, along with the different social-comparative targets people consider to determine their relative social position, suggest that our SSS and PRD measures are not simply tapping the same underlying construct.

In Study 6, we revisited the relative predictive utility of the SES ladder measure and full five-item PRDS by asking a subset of participants from Study 3 to complete the SES ladder, PRDS, and perceived stress scale again after 6 weeks. This approach allowed us examine whether (a) the PRDS is, in fact, a more reliable measure than the SES ladder in terms of test-retest reliability (and therefore possibly explains its superior predictive validity); and (b) changes in PRD and SSS are associated with changes in perceived stress over a 6 week period. Using a change score approach (i.e., where changes in stress are regressed onto changes in PRD, SSS, and objective SES indicators) further addresses the issue of response biases (which are assumed to be time-invariant, e.g., chronic negative affectivity, personality) confounding a measure because such biases are controlled, or zeroed out, within the analysis (i.e., a general negative thinking style that might plague the PRDS more than the SES ladder is assumed to exist across time waves as a chronic individual difference; see Liker et al., 1985).

### Methods

#### Participants

We invited participants who completed our survey in Study 3 through Prolific Academic to complete a follow-up survey 6 weeks later (*N* = 118; *M*_age_ = 29.84, *SD*_age_ = 9.99; % women = 52%). There were no significant differences between those who did and did not complete the second survey through Prolific Academic in terms of SSS, PRD, stress, age, income, gender, and years of education at Time 1 (all *p*s > 0.12).

#### Procedure and measures

Participants first completed the SSS ladder measure and PRDS in a random order. Next, they completed the 10-item Perceived Stress Scale (Cohen and Williamson, 1988) and reported their annual household income and years of education as in Study 3.

### Results and discussion

Shown along the diagonal in Table 9, the SSS ladder measure and PRDS showed acceptable and comparable test-retest reliability across 6 weeks (if anything, test-retest reliability was slightly higher for the SSS measure than the PRDS).

**Table 9:** **Descriptive statistics and intercorrelations for measures in Study 6**.

**Measures**	***Mean* (*SD*)**	**1**	**2**	**3**	**4**	**5**
1. ΔSSS	−0.04 (0.97)	(0.82)				
2. ΔPRDS	0.03 (0.72)	−0.08	(0.75)			
3. ΔIncome	−0.58 (3.19)	−0.14	0.05	(0.72)		
4. ΔEducation	−0.14 (1.46)	−0.07	0.04	−0.04	(0.86)	
5. ΔPerceived Stress	−0.05 (0.51)	−0.04	0.37^**^	−0.07	−0.02	(0.78)

*SSS, Subjective Socioeconomic Status; PRDS, Personal Relative Deprivation Scale; Δ, change. Values along the diagonal depict test-retest reliabilities across the 6 weeks (Pearson product-moment correlations). Higher values indicate more of each construct at Time 2 (T2–T1)*.

** *p < 0.01*.

We examined whether changes in SSS and PRD over the 6 weeks were related to changes in perceived stress over the same period. These analyses were conducted using change scores (T2–T1) for each variable. Shown in Table 9, only changes in PRD correlated significantly with changes in stress. A multiple regression analysis regressing change scores for stress onto change scores for SSS, PRD, income, and education revealed that only changes in PRD accounted for significance incremental variance in changes in perceived stress (see Table 3). A dominance analysis established general dominance of changes in PRD over changes in SSS for the prediction of changes in stress (reproducibility = 99.5%).

## General discussion

The present studies suggest that self-reported health indicators are, by and large, better predicted by PRD than by SSS. As in previous research, SSS significantly correlated with a wide range of health-related measures, including global physical health, perceived stress, sleep quality, mental health impairment, depression, negative affect, and resentment. However, after controlling for PRD, SSS accounted for significant incremental variability in only 5 of the 16 criterion measures we employed across studies. PRD remained a significant predictor of all but one of these measures while controlling for SSS, income, and education. What is more, dominance analyses showed that PRD established general dominance over SSS with a high degree of confidence for the prediction of 13 of the 16 outcome measures we employed across studies, whereas dominance of SSS over PRD was never established. Studies 4–6 established that the superiority of PRD over SSS in predicting mental and physical health is likely not due to differences in the psychometric properties of the measures (e.g., test-retest reliability).

Despite this pattern of findings, it is important to note that, consistent with several previous studies, in two of our studies SSS accounted for significant unique variance in the self-rated health item. Indeed, a multiple regression analysis of the standardized and aggregated data across all of our studies that measured self-rated health (Studies 1, 2, 3, and 5; total *N* = 1523) showed that SSS was a significant predictor of self-rated health over and above PRD, income, and education (β = 0.11, *sr*^2^ = 0.008, *p* < 0.001, *GDW* = 0.035; overall model *R*^2^ = 0.115). Thus, confirming previous research, where people position themselves on a subjective SES measure contributes to self-rated health, even above PRD and objective SES indicators. Nonetheless, PRD was also a unique predictor of self-rated health in this analysis (β = −0.21, *sr*^2^ = 0.033, *p* < 0.001, *GDW* = 0.055; reproducibility of PRD dominating SSS = 80.4%). Further, exploratory moderated regression analyses of these collated data suggest that the relationship between PRD and self-rated health is not significantly moderated by SSS (*p* = 0.38), income (*p* = 0.20), or education (*p* = 0.34), suggesting the possibility that higher PRD contributes to poorer self-rated health even among individuals who are subjectively or objectively wealthy.

Why is PRD a better predictor than the “SES ladder”? One explanation is that the social reference group identified by the MacArthur SSS scale is not the only (or perhaps even most) relevant to mental and physical health. Just as SSS captures a person's self-perceived status in a way that is imperfectly correlated with their absolute wealth, so the “SES ladder” itself may fail to identify the reference groups that are most relevant to the social comparisons that influence health: An American might have a sense of his or her position relative to the rest of the US population but if they primarily compare themselves with their co-workers, friends, and neighbors, then their sense of deprivation may be largely unrelated to their position on the national “SES ladder”; research suggests that such comparison with “similar others” is more likely than comparison with wider society (see e.g., Clark and Senik, 2010), and the PRDS explicitly taps into people's sense of deprivation compared to others who are “like them” (Callan et al., 2011).

A second reason for the relative success of the PRDS is that it assesses the sense of dissatisfaction and resentment engendered by unfavorable comparisons (Smith et al., 2012). People differ in their tendency to compare themselves to others (e.g., Gibbons and Buunk, 1999) and these differences are associated with feelings of relative deprivation (Buunk et al., 2003; Callan et al., 2015). Two people may have the same self-perceived social status but have very different reactions to it, and the PRDS, unlike SSS, explicitly assesses people's sense of privilege, resentment, dissatisfaction, and deprivation. These responses are likely to be what evokes negative responses such as stress which, in turn, may underlie many health outcomes, and our mediation analyses provide initial support for this idea. A related possibility is that health-relevant self-perceived status is partly based on status indicators that are distinct from SSS, such as the richness of one's social circle. By including items that assess a more general sense of relative success, the PRDS may capture relevant dimensions that are missed by a narrower focus on conventional SES indicators. Indeed, the PRDS, compared to the SSS measure, explicitly allows respondents to define (a) their own relevant comparative targets and (b) the dimensions on which they make their comparisons. Therefore, researchers interested in the role that subjective status plays in health might in future consider assessing PRD (e.g., with the PRDS) along with other measures of subjective status (e.g., an SES ladder) to gain a fuller understanding of the relations among subjective social status and health.

The present findings therefore support the broad idea that subjective relative status is an important predictor of health, but suggest some refinement to how this relationship is conceptualized and measured. They also have potential policy implications (Smith and Huo, 2014); reducing wealth inequality has been heralded as a way to improve a nation's health (Wilkinson and Pickett, 2009), but such change may be ineffective unless accompanied by a reduction in people's feelings of resentment and injustice—feelings whose origins will be more complex than one's distance from the top/bottom of an SES ladder.

One limitation of the present studies is the use of cross-sectional designs. Although the primary purpose of these studies was to test the relative predictive utility of PRD and SSS for self-rated health by closely following the research designs and data analytic strategies of previous studies linking SSS to health (which were also cross-sectional), the causal relation between PRD and ill-health remains to be explicated. Despite this limitation, experimental research has provided evidence that adverse social comparisons with similar others causally influence the psychosocial vulnerabilities (e.g., negative affect) hypothesized to mediate the effects of SSS/PRD on health (e.g., Walker, 1999; Callan et al., 2008, 2011). For example, using a false feedback procedure, Callan et al. (2008) found that participants who were led to believe that they had less discretionary income than other psychology students reported greater resentment, dissatisfaction, and a sense of unfairness than participants who believed that their discretionary income was roughly the same as their peers. Nonetheless, an important avenue for future research will be to examine the longitudinal associations between PRD, psychosocial vulnerabilities and health, including whether and how PRD affects ill-health and how ill-health can feedback to affect PRD over time (cf. Schmitt et al., 2010).

Another limitation of the present studies is that they employed self-reported health indicators. Although the measures we used have good external validity, it will be important to see whether our findings generalize to physical health indicators over time, such as blood pressure and susceptibility to infection (e.g., Adler et al., 2000; Cohen et al., 2008). Moreover, because the average age of our participants across our studies was relatively young, future research should aim to investigate the relative contribution of SSS and PRD to the functional decline in older adults (Chen et al., 2012). In addition, the current results lend impetus to efforts to explain individual differences in PRD (Smith et al., 2012), and a key direction for future work will be to examine the psychological processes that underlie people's beliefs about their relative deprivation (e.g., Boyce et al., 2010). Finally, it will be important to probe further the mechanisms by which PRD engenders health outcomes, to establish the specific psychological, behavioral, and physiological consequences of PRD that connect the feeling of deprivation to particular health outcomes.

## Funding

This research was funded by a grant from the Leverhulme Trust (RPG-2013-148).

### Conflict of interest statement

The authors declare that the research was conducted in the absence of any commercial or financial relationships that could be construed as a potential conflict of interest.

## Footnotes

^1^These studies were conducted with the approval of the University of Essex Faculty Research Ethics Committee. All participants gave consent prior to participating. For each study, we recruited participants based on the sample size required to obtain 80% power (usually higher) to detect “medium” effect sizes (*f*^2^ = 0.06) in our multiple regression analyses. The minimum required sample sizes were fixed ahead of data collection, but the final sample sizes were not completely pre-determined due to the vagaries of excessive sign-ups, incomplete data, and removal of participants who completed the survey a second time or failed an attention check.

^2^For consistency with earlier work, we used an ordinal coding of income responses (e.g., 1–8; Kraus et al., 2013) and level of education (e.g., 1–4) for our multiple regression analyses across studies. The results were virtually identical when we coded income responses using the category mid-points (with the value for the open-ended top category being the median-based estimator described by Parker and Fenwick, 1983) and education with three effect coded vectors representing the four possible categories of educational attainment.

^3^Including age and gender as predictors in these analyses yields the same patterns of results for PRD and SSS across studies.

^4^Three participants provided years of education that were not possible given their reported age. We recoded these values to the most post-16 years of education they could have achieved given their age. The results are virtually identical if we leave these values as reported or remove these participants entirely. We used mean replacement for two additional participants who did not provide values for education, which again made virtually no difference to the results (vs. removing them list-wise).
